# WHODAS 2.0-BO: normative data for the assessment of disability in older adults

**DOI:** 10.11606/S1518-8787.2019053000586

**Published:** 2019-01-18

**Authors:** Michele Lacerda Pereira Ferrer, Monica Rodrigues Perracini, Flávio Rebustini, Cassia Maria Buchalla

**Affiliations:** I Universidade São Francisco. Faculdade de Fisioterapia. Bragança Paulista, SP, Brasil; II Universidade de São Paulo. Faculdade de Saúde Pública. Programa de Pós-Graduação em Saúde Pública. São Paulo, SP, Brasil; III Universidade Cidade de São Paulo. Programa de Pós-Graduação em Fisioterapia. São Paulo, SP, Brasil; IV Universidade Estadual de Campinas. Faculdade de Ciências Médicas. Programa de Pós-Graduação em Gerontologia. Campinas, SP, Brasil; V Universidade Estadual Paulista “Júlio de Mesquita Filho”. Rio Claro, SP, Brasil; VI Universidade de São Paulo. Escola de Artes, Ciências e Humanidades. Programa de Pós-Graduação em Gerontologia. São Paulo, SP, Brasil; VII Universidade de São Paulo. Faculdade de Saúde Pública. Departamento de Epidemiologia. São Paulo, SP, Brasil

**Keywords:** Aged, Disability Evaluation, International Classification of Functioning, Disability and Health, Comorbidity, Socioeconomic Factors, Surveys and Questionnaires, standards, Idoso, Avaliação da Deficiência, Classificação Internacional de Funcionalidade, Incapacidade e Saúde, Comorbidade, Fatores Socioeconômicos, Inquéritos e Questionários, normas

## Abstract

**OBJECTIVE:**

To examine the normative data of WHODAS 2.0-BO for older Brazilians (World Health Disability Assessment Schedule – Brazilian version for older people) and its distribution according to sex, age, health, subjective health perception, performance in a mobility test and presence of chronic diseases and depression.

**METHODS:**

Cross-sectional study, with 350 participants with 60 years of age or older, men and women, patients of a geriatric specialized center for medical consultations or rehabilitation. The older adults were evaluated using a semi-structured questionnaire containing demographic and clinical data (WHODAS 2.0-BO) and the geriatric depression scale (GDS), having been subsequently subjected to a mobility test (Timed Up and Go). The data were analyzed via their distribution in percentiles of the population and via analysis of variance.

**RESULTS:**

Two-hundred and sixty-six (76%) participants were women, and the average age was 71.8 (DP = 6.7) years old. The average score in WHODAS 2.0-BO was 4.3 (DP = 5.2) points, the highest value found having corresponded to 33 points. The average time for the Timed Up and Go test was 10.0 (SD = 3.2) seconds. About 30% of the older adults did not report any difficulties in the tasks evaluated by WHODAS 2.0-BO and half of the sample scored up to two points.

**CONCLUSIONS:**

A score corresponding to 12 points in the 90 percentile on a scale from zero to 40 was observed, which suggests severe disability. The score in WHODAS 2.0-BO increased with the advance in age, as well as in the presence of comorbidities, negative health perception, depression, high blood pressure, visual and hearing impairment and mobility impairment.

## RESUMO

**OBJETIVO:**

Examinar os dados de normatização do WHODAS 2.0-BO para idosos brasileiros (*World Health Disability Assessment Schedule – Brazilian version for older people*) e sua distribuição de acordo com sexo, idade, percepção subjetiva de saúde, desempenho em teste de mobilidade e presença de doenças crônicas e depressão.

**MÉTODOS:**

Estudo transversal, com 350 participantes com 60 anos ou mais, homens e mulheres, atendidos em um centro de referência secundário para consultas médicas ou de reabilitação. Os idosos foram avaliados por meio de um questionário semiestruturado, contendo dados sociodemográficos e clínicos, o WHODAS 2.0-BO e a escala de depressão geriátrica (EDG), e submetidos a um teste mobilidade, o *Timed Up and Go*. Os dados foram analisados por sua distribuição em percentis na população e por análise de variância.

**RESULTADOS:**

Duzentos e sessenta e seis (76%) participantes eram mulheres, a idade média foi de 71,8 (DP = 6,7) anos. O escore médio do WHODAS 2.0-BO foi de 4,3 (DP = 5,2) pontos, sendo o maior valor encontrado 33 pontos. O tempo para o *Timed up and Go* foi de 10,0 (DP = 3,2) segundos. Cerca de 30% dos idosos não relataram nenhuma dificuldade nas tarefas avaliadas pelo WHODAS 2.0-BO e metade da amostra alcançou até dois pontos.

**CONCLUSÕES:**

Observou-se um escore de 12 pontos no percentil 90 em uma escala de zero a 40, o que sugere incapacidade grave. O escore do WHODAS 2.0-BO aumentou com o avançar da idade, bem como na presença de comorbidades, de percepção subjetiva de saúde ruim, de depressão, de hipertensão arterial, de dificuldade para enxergar e escutar e de alteração da mobilidade.

## INTRODUCTION

The population's ageing is today a reality not only in developed countries, but also in developing countries like Brazil. Although a decrease in the prevalence of functional disability in the older population has been observed in recent decades in high-income countries, this scenario is still intangible for most older adults^1^ ^,^ ^2^. The increase in life expectancy seen in recent years has not been reflected in an increase in healthy ageing, recognized as the welfare provided by the maintenance of functional capacity in old age, especially in developing countries. The increased prevalence of chronic diseases and unhealthy lifestyles and health inequities have considerable impact on this population, verified by the increase in disability indicators^1^ ^,^ ^3^.

Functional evaluation measures that can identify not only impairments to the activities of daily living, but also to the restriction of participation in the community, are thus of great relevance. The early identification of loss of functionality assists the planning of preventive strategies, avoiding the aggravation or the appearance of greater functional disabilities^4^. Disability must be understood as a comprehensive, dynamic and multidimensional concept^5^.

The International Classification of Functioning, Disability and Health (ICF-WHO) determines the individuals’ function and health aspects, independently from a specific assessment instrument. It describes the functioning and disability related to health conditions and to the functions of the organs or systems and structures of the body, in addition to the limitations of activities and social participation in the individual's life environment^6^. An important part of classification refers to activities and social participation. Activity is defined as the execution of a task or action by an individual. It represents the subjective perspective of functioning while its negative side refers to the activity limitations, defined as the difficulty an individual may face when performing it. Participation is defined as the involvement in a life situation, i.e., it represents the social perspective of functioning, and its negative side is the restriction of participation, due to the problems that an individual may experience in the involvement with these situations^6^.

Disability is a generic term used by ICF to translate the negative aspects of the interaction between an individual with a certain health condition and the contextual factors, in a dynamic relationship^6^. However, due to its multidimensionality, ICF still cannot identify disability using a single instrument or evaluation. For the assessment of disability in activities and participation, WHO proposed WHODAS 2.0 (World Health Disability Assessment Schedule). It is a generic instrument, developed from a set of items of ICF and that can be applied to different populations both in clinical contexts, to measure the impact of a given intervention, and in the population context of epidemiological studies^7^ ^,^ ^8^.

WHO published WHODAS 2.0 (2010) as an update of version WHODAS II that was tested in multicenter studies. WHODAS 2.0 has been translated into 47 languages and dialects and used in different populations^7^. The instrument is presented in three versions: one with 36 items, another summarized into 12 items and a last one combining 12+24 items. The full version, with 36 items, has been the most widely used and studied in relation to its invariance and psychometric properties. Version 12+24 is a simple hybrid of the versions with 12 and 36 items. If the 12 initial items are answered positively, 24 additional questions are applied, completing the 36-item version. In the case of negative answers to the 12 initial questions, the others are not applied^8^. The 12-item version is indicated for use in population studies or in situations where time does not allow performing a more detailed assessment. There is no consensus in the literature about the dimensionality of the 12-item version when analyzing its psychometric properties in different samples^8^ ^,^ ^9^. The same inconsistency is observed when the instrument is applied to older adults^9^ ^,^ ^10^.

A construct validation study of the 12-item version applied to older Brazilians showed that 10 items were sufficient to measure disability^a^. However, the meaning of the scores has not yet been presented. As important as developing or adapting an instrument is interpreting its scores and assigning them meaning, which is possible via normalization^11^.

Normalization refers to interpretation standards in relation to a score assigned to an individual in a particular test, indicating his relative position in the normative sample and comparing his performance to other people's^12^. As the World Health Disability Assessment Schedule – Brazilian version for older people (WHODAS 2.0-BO) examines different dimensions of activities and social participation and considering these influence the well-being and functionality of older adults, a normalization of its score is important. It will allow the comparison of a score between individuals or in samples with similar profiles, providing a better analysis both from a clinical and from an epidemiological point of view.

Normative data of the WHODAS 2.0-BO version for older Brazilians are necessary for the use of the instrument to be viable, for it to have clinical significance and for the results obtained to be comparable. It is also important to identify whether this instrument can recognize factors associated with the disability of older adults in the community so that preventive measures may be proposed^4^.

This study had as aim examining the normative data of WHODAS 2.0-BO among seniors in the community and their distribution according to sex, age, health, subjective health perception, performance in a mobility test, presence of chronic diseases and depression.

## METHODS

Cross-sectional study, with secondary data sources from a sample selected by convenience in the study “*Adaptação transcultural para o português brasileiro e validação concorrente do* Incidental and Planned Exercise Questionnaire *(IPEQ) para pessoas idosas*” [Cross-cultural translation into Brazilian Portuguese and concurrent validation of the Incidental and Planned Exercise Questionnaire (IPEQ) for older adults]^b^. This sample included interviews with 350 older people of the community, patients of a center of reference for older adults, in São Paulo, state of São Paulo, in the period from September 2013 to May 2014. This location is a secondary care service with experts in the field of Geriatrics and Gerontology, featuring multidisciplinary care. The older adults, of both sexes, aged over 60 years old, answered WHODAS 2.0-BO while waiting for a medical consultation and for physiotherapy and occupational therapy sessions.

The older adults with cognitive impairment according to their scores in the mini-mental state examination (MMSE), < 19 for illiterate older adults and ≤ 23 for those with some level of education, with mobility and orthostatic disabilities (even with the support of aid devices), and/or with severe hearing or language impairment that hindered communication, were excluded from the study. All the older adults who agreed to participate in the study signed the informed consent form (CAAE 09091712.3.0000.0064).

### Instruments

The older adults answered the WHODAS 2.0 questions about the difficulties faced by the individual in the last 30 days in an interview^7^. For this study, the WHODAS 2.0-BO score was calculated using a simple sum, with the variables’ categories ranging from zero (no problem) to four (severe problem or unable to perform). The total WHODAS 2.0-BO score can vary from zero to 40 points (Table 1).

**Table 1:** Sociodemographic and health characteristics, according to frequency and score in WHODAS 2.0-BO, of the population studied.

Variable		n	%	Score in WHODAS 2.0-BO		p
				Mean (SD)	Median	
Age group (years)						0.025^a^
	60 |-| 69	136	38.9	3.7 (5.2)	2.0	
	70 |-| 79	170	48.6	4.4 (5.1)	3.0	
	80 or more	44	12.6	5.5 (5.2)	4.0	
Gender						0,982^b^
	Male	84	24.0	3.7 (4.0)	3.0	
	Female	266	76.0	4.4 (5.5)	2.0	
Marital status						0.078^a^
	Married	148	42.3	4.0 (5.1)	2.0	
	Single	29	8.3	2.3 (3.5)	1.0	
	Widow/widower	142	40.6	4.7 (5.3)	3.0	
	Divorced	31	8.9	5.1 (6.0)	3.0	
Education level						0.003^a^
	Illiterate	13	3.7	10.1 (7.2)	10.0	
	Elementary school	267	76.3	4.2 (4.9)	3.0	
	High school	51	14.6	4.0 (5.4)	2.0	
	Higher education	19	5.4	3.5 (4.8)	2.0	
Lives alone						0.531^b^
	Yes	101	28.9	3.9 (4.6)	2.0	
	No	248	71.1	4.4 (5.4)	3.0	
Diseases reported						
	CVA	26	7.4	5.1 (4.8)	4.0	0.061^b^
	Parkinson's disease	11	3.1	5.1 (4.2)	5.0	0.244^b^
	Depression	83	23.7	6.3 (6.6)	3.0	< 0.001^b^
	Arterial hypertension	243	69.4	4.7 (5.1)	3.0	0.001^b^
	Diabetes	144	41.1	4.4 (4.9)	3.0	0.190^b^
	Urinary incontinence	76	21.7	5.2 (5.8)	4.0	0.061^b^
Uses mobility aid devices						
	Yes	40	11.4	9.0 (5.5)	8.5	< 0.001^b^
	No	310	88.6	3.7 (4.8)	2.0	
Visual impairment						< 0.001^b^
	Yes	182	52.0	5.0 (5.3)	3.0	
	No	168	48.0	3.5 (5.0)	2.0	
hearing impairment						0.040^b^
	Yes	112	32.0	5.0 (5.3)	3.0	
	No	238	68.0	3.9 (5.1)	2.0	
Subjective health perception						< 0.001^a^
	Very good	51	14.6	1.0 (2.0)	0.0	
	Good	142	40.6	2.6 (3.0)	2.0	
	Average	131	37.4	6.1 (5.8)	5.0	
	Poor	19	5.4	9.2 (6.5)	7.0	
	Very poor	7	2.0	12.3 (10.1)	9.0	
GDS						< 0.001^b^
	Up to 5 points	292	83.4	3.4 (4.2)	2.0	
	Above 5 points	58	16.6	8.4 (7.3)	7.0	
TUGT						
	0–10 seconds	212	60,6	2.6 (3.9)	2.0	
	11 seconds or more	138	39.4	6.9 (5.8)	5.5	< 0.001^b^

CVA: cerebrovascular accident; GDS: geriatric depression scale; TUGT: timed up and go test

a p ≤ 0.05 by the Kruskal-Wallis test.

b p ≤ 0.05 by the Mann-Whitney test.

The short Geriatric Depression Scale – GDS, with 15 items, was applied. The score obtained by the negative answers ranges from zero to 15, results exceeding five points being indicative of depressive states^13^; and the Timed Up and Go test (TUGT), which evaluates mobility by analyzing the individual's performance when getting up from an armed chair, walking three meters, turning, returning and sitting again as quickly as possible, in a safe way^14^. An attempt at familiarization is made and the time is computed in seconds.

The older adults were questioned regarding their subjective perception of health, how much the difficulties reported interfered with their daily routine, and which diseases had been diagnosed by a doctor in the past three months. Demographic data such as age, sex, income, education and occupational activity were also obtained.

### Statistical Analysis

The characteristics of the sample are presented in frequency for the nominal variables and in mean, median and standard deviation for the numeric variables. The distribution of the WHODAS 2.0-BO score was calculated for percentiles 25, 50, 75 and 90. To verify the difference of the mean WHODAS 2.0-BO score according to the qualitative variables of interest, the Mann-Whitney test was used for the dichotomous variables and the Kruskal-Wallis test was used for variables with more than two categories, due to the non-parametric distribution of the data. For the analysis of the sample's profile according to the cross-sections, the analysis of percentiles was used^12^.

## RESULTS

Among the 350 older adults interviewed, 76% (266) were female, with the following averages: 71.8 (SD = 6.7) years of age; 5.8 (SD = 3.8) years of education; R$1.050,00 income (SD = 654.7); 25.8 (SD = 3.1) points in the MMSE. With regard to disability, the older adults’ score in WHODAS 2.0-BO was 4.3 (SD = 5.2) points, and they completed the TUGT in 10 (SD = 3.2) seconds.

The older adults evaluated reported 2.3 chronic diseases on average and only 10.6% (n = 37) did not report any disease (Table 1).

When analyzing the distribution of the WHODAS 2.0-BO score in the sample, we found that almost a third of it (n = 102) reported no difficulty in the tasks addressed. Half of the sample scored up to two points and, in the 90 percentile, 12 points, on a scale from zero to 40 (Table 2). The maximum score was 33. The frequency of the respondents’ answers for each category is presented in Table 3.

**Table 2:** Distribution of the WHODAS 2.0-BO score in the study population.

Score	n	%	Accumulated %
0.0	102	29.1	29.1
1.0	29	8.3	37.4
2.0	45	12.9	50.3
3.0	32	9.1	59.4
4.0	24	6.9	66.3
5.0	19	5.4	71.7
6.0	14	4.0	75.7
7.0	20	5.7	81.4
8.0	9	2.6	84.0
9.0	5	1.4	85.4
10.0	8	2.3	87.7
11.0	4	1.1	88.9
12.0	10	2.9	91.7
13.0	5	1.4	93.1
14.0	5	1.4	94.6
15.0	2	0.6	95.1
16.0	4	1.1	96.3
17.0	2	0.6	96.9
18.0	1	0.3	97.1
19.0	1	0.3	97.4
20.0	4	1.1	98.6
21.0	1	0.3	98.9
22.0	1	0.3	99.1
23.0	1	0.3	99.4
24.0	1	0.3	99.7
33.0	1	0.3	100.0
Total	350	100.0	

**Table 3:** Distribution of the participants’ answers to WHODAS 2.0-BO, n (%), according to each item of the instrument.

Difficulties in the last 30 days to	None	Mild	Medium	Severe	Extreme/I am unable to
1 – Stand for periods as long as or longer than 30 minutes	217 (62.0)	57 (16.3)	59 (16.9)	14 (4.0)	3 (0.9)
2 – Do your daily chores	244 (69.7)	53 (15.1)	39 (11.1)	13 (3.7)	1 (0.3)
3 – Learn a new task, for example, how to get to a new place	243 (69.4)	52 (14.9)	42 (12.0)	7 (2.0)	6 (1.7)
4 –Difficult to engaging (participating) in the community's activities (for example, festivities, religious activities and others) in the same way as everyone else	298 (85.1)	33 (9.4)	15 (4.3)	4 (1.1)	0 (0.0)
5 – been emotionally affected by your health problems	261 (74.6)	47 (13.4)	29 (8.3)	11 (3.1)	2 (0.6)
6 – Walk a great distance, as for example, one kilometer (about 10 blocks)	170 (48.6)	51 (14.6)	72 (20.6)	43 (12.3)	14 (4.0)
7 – Wash your whole body	310 (88.6)	21 (6.0)	18 (5.1)	0 (0.0)	1 (0.3)
8 – Dress yourself	287 (82.0)	36 (10.3)	24 (6.9)	3 (0.9)	0 (0.0)
9 – Maintain a friendship	303 (82.6)	31 (8.9)	15 (4.3)	1 (0.3)	0 (0.0)
10 – Work on a daily basis	289 (82.6)	37 (10.6)	19 (5.4)	4 (1.1)	1 (0.3)

We observed that the distribution of the WHODAS 2.0-BO score in percentiles increased with the advance of age, in the presence of three or more chronic diseases and in the case of very significant interference of the difficulties in their daily routine. Women differed from men with higher scores, from the 75 percentile up (Table 4). However, the results of the non-parametric Mann-Whitney U test showed no statistically significant difference between the score in WHODAS 2.0-BO and sex (p = 0.982).

**Table 4:** WHODAS-BO score distribution in percentiles (weighed average), with 10 items, according to age, sex, number of chronic diseases and perception of interference of functional difficulties in daily life.

Variable		Percentiles			
		25	50	75	90
Total sample – Sex		0.0	2.0	6.0	12.0
	Men	0.2	3.0	5.0	9.0
	Women	0.0	2.0	7.0	12.0
Age group (years)					
	60–69	0.0	2.0	5.0	10.3
	70–79	0.0	3.0	7.0	12.0
	80 or more	2.0	4.0	7.0	14.5
Number of diseases					
	0	0.0	0.0	2.0	7.0
	1–2	0.0	2.0	5.0	10.0
	3 or more	2.0	4.0	8.0	14.0
How much the difficulties interfere with your life					
	Nothing	2.0	3.0	4.0	7.0
	Mildly	0.0	1.0	3.0	5.0
	Some	2.0	4.0	7.5	13.6
	Very much	6.0	10.0	14.0	18.3
	Totally	7.5	11.0	17.0	–

Among the age groups, statistically significant differences were observed in the WHODAS 2.0-BO score (Kruskal-Wallis test, p = 0.025), as well as in the number of chronic diseases, subjective perception of health and interference of the difficulties reported in daily life (p < 0.001) (Box).

**Box:** WHODAS 2.0-BO (*Brazilian version for older people*)*.

In the past 30 days, how muchdifficulty did you have in:					
1 – Standing for long periods such as 30 minutes?	None	Mild	Moderate	Severe	Extreme/Can not do
2 – Taking care of your household responsabilities?	None	Mild	Moderate	Severe	Can not do
3 – Learning a new task, for example, how to get to a new place?	None	Mild	Moderate	Severe	Can not do
4 – Joining in community acitivities(for example, festivities, religious activities and others) in the same way as everyone else?	None	Mild	Moderate	Severe	Can not do
5 – Have you been emotionally affected by your health problems?	None	Mild	Moderate	Severe	Can not do
6 – Waling a long distance such asa kilometer (about 10 blocks)?	None	Mild	Moderate	Severe	Can not do
7 – Washing your whole body?	None	Mild	Moderate	Severe	Can not do
8 –getting dressed?	None	Mild	Moderate	Severe	Can not do
9 – Maintaiing n a friendship?	None	Mild	Moderate	Severe	Can not do
10 – your day-to-day work?	None	Mild	Moderate	Severe	Can not do

* Michele LPF. O impacto dos fatores ambientais na incapacidade funcional de idosos: a importância de políticas públicas que valorizem o Aging in place [These]. São Paulo: Faculdade de Saúde Pública; 2018 [cited 2018 Dec 16]. https://doi.org/10.11606/T.6.2018.tde-23032018-094707

## DISCUSSION

When examining the normative data of WHODAS 2.0-BO among older adults in the community, we identified that there was no statistically significant difference between men and women^15^ ^,^ ^16^. The disability score, measured by WHODAS 2.0-BO, was higher for those who were older, illiterate, with presence of three or more chronic diseases, especially when these were associated with depression and hypertension. It was also higher among those who needed to use mobility aid devices, who required more than 10 seconds to complete the TUGT, and those who had trouble seeing or hearing. In addition, the subjective perception of poor or very poor health and the perception of impairments in routine activities due to the difficulties in the tasks evaluated were strongly associated with a higher score in WHODAS 2.0-BO.

We noted that the WHODAS 2.0-BO score featured a high concentration of “no difficulty” answers (score 0), which corroborates data from other studies that analyzed the distribution of scores in the 12-item version of WHODAS^15^ ^,^ ^17^. This characteristic of the instrument reinforces its ability to measure disability, and not functioning, since it does not have the necessary specificity to indicate small differences between individuals with minimal difficulties or lower disability^9^ ^,^ ^18^.

We proposed, in this study, cut-off scores based on the distribution by percentiles in the general sample. Older adults with high level of disability are expected to be found in the 90 percentile; in this study, the respondents scored above 12 in WHODAS 2.0-BO and, therefore, may be classified with severe disability. Moderate disability would be found among those who score between six to 11, and mild disability, among those who score between two and five.

When using the 12-item version, simple scoring (which may feature sums that range between zero – no disability – to 48 points – with full disability) or scoring based on the item-response theory (0–100), produce similar scores without substantial changes in the interpretation of the results^17^. In the normative population data of the 12-item version submitted by WHO, 50% of the population scored zero (no difficulty). The 90 percentile of the population corresponded to those who scored 17 (in a metric from zero to 100)^7^. In another study, which used normative data of the Australian population^17^, 45% of the sample scored zero (no difficulty). People who were more likely to experience disability (90 percentile) scored above 10 (scale of 0–48 points). The scores increased with age (when controlled by sex), which suggests age-based scoring: for people with 65 to 74 years of age, the average score was 3.7 (SD = 5.5) points, indicative of mild disability; for those with 75 to 85 years of age, the average was 5.7 (DP = 7.1) points, indicating moderate disability. Moreira et al.^19^, who also used a simple sum to calculate the results of a research with 144 older adults in Portugal, found 2.5 (SD = 4.45) points as average value of the 12-item WHODAS 2.0, which also increased with age. The older adults with 74 to 85 years of age scored 3.4 (SD = 3.2) on average.

The increase in the WHODAS 2.0-BO score was associated with increased comorbidities, in particular those related to mental (depression) and physical health. This can be attributed not only to the design of the instrument which includes the assessment of tasks that consider the emotional and physical performance issue, but also to the evidence that such diseases are more crippling for some older adults than for others^20^ ^-^ ^22^.

The WHODAS 2.0-BO score increased with age, indicating that this increase is associated with disability^23^. This finding corroborates other Brazilian studies that associate the weight of ageing with functioning. It is important to note, however, that most of these studies were based on the evaluation of dependency for instrumental and basic activities of daily living^5^ ^,^ ^21^ ^,^ ^24^ ^,^ ^25^. We did not find any population study that examined the disability of older Brazilians using WHODAS 2.0.

We observed that the older adults who perceived their health as very poor obtained high scores in WHODAS 2.0-BO (12 points on average), reinforcing the findings that indicate the self-assessment of health as a good predictor of mortality and disability among older people. The self-assessment of health reflects the integrated perception between the physical, psychological and social dimensions of the individual's health, reinforcing WHO's concept of health in relation to the importance of multi-dimensional well-being, and not merely the absence of diseases^26^ ^-^ ^28^. In this study, the presence of at least one chronic disease was indicated by 89% of the sample, but only 7.4% of it assessed their own health as poor or very poor^26^.

Difficulty to move, evidenced by the performance in the TUGT and by the need to use mobility aid devices, was strongly associated with a higher score in WHODAS 2.0-BO. Mobility issues are often mentioned as the first signs of disability, frailty and falls^29^ ^,^ ^30^. These data are so important to the functioning of older adults that they have already been identified as predictors of mortality, as found in the longitudinal 11-yearlong study by Bergland et al. in 2017^30^.

This study has limitations related to the size and representativeness of the sample, which was chosen by convenience and did not include institutionalized older adults (who would probably have a higher level of disability). The criteria were also not validated due to the absence of a gold standard instrument in the collection of data that could be used for this purpose. Future invariance analyses must be performed with larger samples to verify the suitability of the score here suggested. More cross-sectional studies are also needed for comparability with other populations, as well as longitudinal studies for comparability over time.

## CONCLUSION

This study indicated normative scores using percentiles for the application of WHODAS 2.0-BO in older adults of the community. Based on this criterion, older adults who scored above 12 points were considered to have serious disability. However, the importance of analyzing the differences in score according to age group should be considered. The WHODAS 2.0-BO score allowed identifying a significant association between disability and old age, poor subjective health perception, slower performance in the TUGT, hearing and visual impairment and presence of chronic diseases and depression. Early intervention on these risk factors, which are easily detectable, may prevent the advance of disability in older adults.

## Footnotes

a Michele LPF. O impacto dos fatores ambientais na incapacidade funcional de idosos: a importância de políticas públicas que valorizem o Aging in place [These]. São Paulo: Faculdade de Saúde Pública; 2018 [cited 2018 Dec 16]. https://doi.org/10.11606/T.6.2018.tde-23032018-094707

b Lima WP. Adaptação cultural para o português falado no Brasil e validação concorrente do Incidental and Planned Exercise Questionnaire (IPEQ) para pessoas idosas [dissertation]. São Paulo: Universidade Cidade de São Paulo; 2014.

## References

[1] Chatterji S, Byles J, Cutler D, Seeman T, Verdes E (2015). Health, functioning, and disability in older adults: present status and future implications. Lancet.

[2] Lima-Costa MF, Facchini LA, Matos DL, Macinko J (2012). Mudanças em dez anos das desigualdades sociais em saúde dos idosos brasileiros (1998-2008). Rev Saude Publica.

[3] Beard JR, Officer A, Carvalho IA, Sadana R, Pot AM, Michel JP (2016). The World report on ageing and health: a policy framework for healthy ageing. Lancet.

[4] Andrade KRC, Silva MT, Galvão TF, Pereira MG (2015). Functional disability of adults in Brazil: prevalence and associated factors. Rev Saude Publica.

[5] Goujon N, Devine A, Baker SM, Sprunt B, Edmonds TJ, Booth JK (2014). A comparative review of measurement instruments to inform and evaluate effectiveness of disability inclusive development. Disabil Rehabil.

[6] Organização Pan-Americana da Saúde (2003). CIF: Classificação Internacional de Funcionalidade, Incapacidade e Saúde.

[7] Organização Mundial da Saude (2015). Avaliação de saúde e deficiência: manual do WHO Disability Assessment Schedule 9 (WHODAS 2.0).

[8] Federici S, Bracalenti M, Meloni F, Luciano JV (2017). World Health Organization Disability Assessment Schedule 2.0: an international systematic review. Disabil Rehabil.

[9] Gaskin CJ, Lambert SD, Bowe SJ, Orellana L (2017). Why sample selection matters in exploratory factor analysis: implications for the 12-item World Health Organization Disability Assessment Schedule 2.0. BMC Med Res Methodol.

[10] Sousa RM, Dewey ME, Acosta D, Jotheeswaran AT, Castro-Costa E, Ferri CP (2010). Measuring disability across cultures: the psychometric properties of the WHODAS II in older people from seven low- and middle-income countries. The 10/66 Dementia Research Group population-based survey. Int J Methods Psychiatr Res.

[11] Hutz CS, Bandeira DR, Trentini CM (2015). Psicometria.

[12] Erthal TC (2009). Manual de psicometria.

[13] Paradela EMP, Lourenço RA, Veras RP (2005). Validation of geriatric depression scale in a general outpatient clinic. Rev Saude Publica.

[14] Alexandre TS, Meira DM, Rico NC, Mizuta SK (2012). Accuracy of Timed Up and Go Test for screening risk of falls among community-dwelling elderly. Rev Bras Fisioter.

[15] Kirchberger I, Braitmayer K, Coenen M, Oberhauser C, Meisinger C (2014). Feasibility and psychometric properties of the German 12-item WHO Disability Assessment Schedule (WHODAS 2.0) in a population-based sample of patients with myocardial infarction from the MONICA/KORA myocardial infarction registry. Popul Health Metr.

[16] Luciano JV, Ayuso-Mateos JL, Aguado J, Fernandez A, Serrano-Blanco A, Roca M (2010). The 12-item World Health Organization Disability Assessment Schedule II (WHO-DAS II): a nonparametric item response analysis. BMC Med Res Methodol.

[17] Andrews G, Kemp A, Sunderland M, Von Korff M, Ustun TB (2009). Normative data for the 12 item WHO Disability Assessment Schedule 2.0. PLoS One.

[18] Saltychev M, Bärlund E, Mattie R, McCormick Z, Paltamaa J, Laimi K (2017). A study of the psychometric properties of 12-item World Health Organization Disability Assessment Schedule 2.0 in a large population of people with chronic musculoskeletal pain. Clin Rehabil.

[19] Moreira A, Alvarelhão J, Silva AG, Costa R, Queirós A (2015). Tradução e validação para português do WHODAS 2.0 – 12 itens em pessoas com 55 ou mais anos. Rev Port Saude Publica.

[20] Virués-Ortega J, Pedro-Cuesta J, Del Barrio JL, Almazan-Isla J, Bergareche A, Bermejo-Pareja F (2011). Medical, environmental and personal factors of disability in the elderly in Spain: a screening survey based on the International Classification of Functioning. Gac Sanit.

[21] Barbosa BR, Almeida JM, Barbosa MR, Rossi-Barbosa LAR (2014). Avaliação da capacidade funcional dos idosos e fatores associados à incapacidade. Cienc Saude Coletiva.

[22] Rodríguez-Blázquez C, Damián J, Andrés-Prado MJ, Almazán-Isla J, Alcalde-Cabero E, Forjaz MJ (2016). Associations between chronic conditions, body functions, activity limitations and participation restrictions: a cross-sectional approach in Spanish non-clinical populations. BMJ Open.

[23] Almazán-Isla J, Comín-Comín M, Damián J, Alcalde-Cabero E, Ruiz C, Franco E (2014). Analysis of disability using WHODAS 2.0 among the middle-aged and elderly in Cinco Villas, Spain. Disabil Health J.

[24] Giacomin KC, Peixoto SV, Uchoa E, Lima-Costa MF (2008). Estudo de base populacional dos fatores associados à incapacidade funcional entre idosos na Região Metropolitana de Belo Horizonte, Minas Gerais, Brasil. Cad Saude Publica.

[25] Alexandre TS, Corona LP, Nunes DP, Santos JLF, Duarte YAO, Lebrão ML (2014). Disability in instrumental activities of daily living among older adults: gender differences. Rev Saude Publica.

[26] Pagotto V, Bachion MM, Silveira EA (2013). Autoavaliação da saúde por idosos brasileiros: revisão sistemática da literatura. Rev Panam Salud Publica.

[27] Lima-Costa MF, Firmo JOA, Uchôa E (2004). A estrutura da auto-avaliação da saúde entre idosos : projeto Bambuí. Rev Saude Publica.

[28] Alves LC, Leite IC, Machado CJ (2010). Fatores associados à incapacidade funcional dos idosos no Brasil: análise multinível. Rev Saude Publica.

[29] Silva AG, Queiros A, Sa-Couto P, Rocha NP (2015). Self-reported disability: association with lower extremity performance and other determinants in older adults attending primary care. Phys Ther.

[30] Bergland A, Jørgensen L, Emaus N, Strand BH (2017). Mobility as a predictor of all-cause mortality in older men and women: 11.8 year follow-up in the Tromsø study. BMC Health Serv Res.

